# Toward virtual bladder: real-time bladder volume monitoring with flexible AuCNT strain sensors

**DOI:** 10.3389/fbioe.2025.1717576

**Published:** 2026-01-05

**Authors:** Youngjun Cho, Yujin Jo, Minseok Kang, Heejae Shin, Jeongmok Cho, HyungHwa Jeong, HyunSuk Peter Suh, ChangSik John Pak, Jeonhyeong Park, Soonchul Kwon, Hongsoo Choi, Jaesok Yu, Hoe Joon Kim, Sanghoon Lee

**Affiliations:** 1 Department of Robotics and Mechatronics Engineering, Daegu Gyeongbuk Institute of Science and Technology, Daegu, Republic of Korea; 2 Department of Plastic and Reconstruction Surgery, Ewha Womans University Medical Center, Seoul, Republic of Korea; 3 Department of Plastic and Reconstructive Surgery, Soon Chun Hyang University Bucheon Hospital, Bucheon-Si, Republic of Korea; 4 Department of Plastic and Reconstructive Surgery, Asan Medical Center, Seoul, Republic of Korea; 5 Department of Smart Convergence, Kwangwoon University, Seoul, Republic of Korea; 6 Department of Biomedical Engineering, University of Massachusetts Amherst, Amherst, MA, United States

**Keywords:** digital twin, bladder strain sensor, AuCNT, bladder dysfunction, closed-loop neuromodulation

## Abstract

Digital twin technology holds considerable potential for personalized diagnostics and treatment of bladder dysfunction, particularly neurogenic conditions such as underactive bladder (UAB). In this study, to address the need for precise monitoring, we introduce a flexible, stretchable strain sensor composed of gold-coated carbon nanotubes (AuCNTs) embedded in Ecoflex. We specifically designed a three-channel configuration to capture anisotropic expansion and evaluated the sensor’s performance using both two-dimensional balloon models and ex-vivo three-dimensional porcine bladder models. As a result, the AuCNT sensor demonstrated high sensitivity, and the three-channel design significantly enhanced spatial accuracy compared to single-channel approaches. Based on these measurements, we created a preliminary “Virtual Bladder” model that provides dynamic, real-time visualization of bladder volume changes. While our current model requires further development to incorporate multimodal data and anatomical variability, it serves as a foundational step towards developing advanced digital twin frameworks and closed-loop neuromodulation systems for bladder dysfunction.

## 1 Introduction

Bladder dysfunction is a significant health issue, increasingly prevalent in aging populations due to chronic diseases (Lee et al., 2019a). Neurogenic bladder, often resulting from nerve damage associated with spinal cord injury, stroke, or multiple sclerosis, manifests as either overactive bladder or underactive bladder (UAB). UAB, characterized by inadequate bladder condition and incomplete emptying, can lead to severe complications, including urinary tract infections, bladder stones, and even kidney damage. Traditional management methods, such as intermittent catheterization or indwelling catheters are invasive, uncomfortable, and carry infection risks, significantly impacting patients’ quality of life. Neuromodulation, involving electrical stimulation to induce bladder contractions, has emerged as a promising alternative (Lee et al., 2019b). However, its effectiveness heavily relies on accurate, real-time bladder volume monitoring, a significant challenge due to impaired sensory feedback in patients with neurogenic bladder.

**FIGURE 1:**
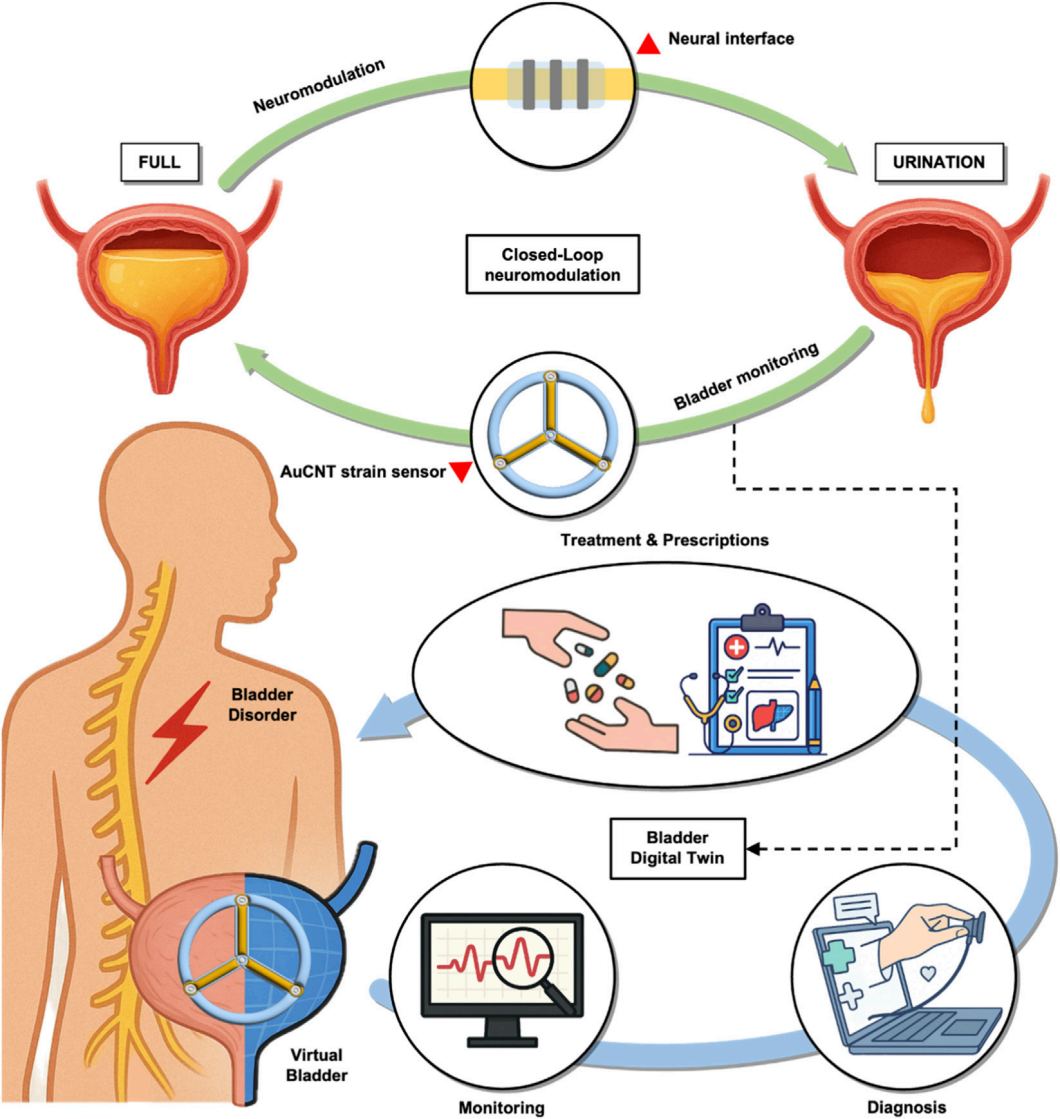
Schematic illustration of closed-loop bladder monitoring and its potential application to a digital twin system using the developed AuCNT-based strain sensor. The developed AuCNT-Ecoflex strain sensor enables real-time monitoring of bladder volume changes, which could support the implementation of a closed-loop control system. By continuously transmitting sensor data to a virtual bladder model, this system may facilitate the development of a digital twin framework for bladder dysfunction, with potential utility in remote diagnosis, adaptive therapy, and personalized treatment strategies.

Recent advances in healthcare technology have created new opportunities to address this gap (Haleem et al., 2023). One such approach may lie in the use of digital twin frameworks, which conceptually involve a real-time, data-driven virtual representation of a physical system (Tao et al., 2022). By integrating continuous sensor data with computational modeling, such systems could enable closed-loop feedback and personalized therapy (Figure 1) (Erol et al., 2020; Singh et al., 2021; Vallée, 2023). In the context of bladder dysfunction, a digital twin might allow for individualized bladder monitoring and adaptive neuromodulation strategies (Singh et al., 2021). However, the realization of such a system requires not only sensitive and biocompatible sensors but also robust algorithms, efficient data processing, and anatomical adaptability, areas that remain underexplored. Therefore, as a starting point of the study, it is essential to develop a stable and non-invasive bladder monitoring system that enables precise neuromodulation (Sun et al., 2023).

The bladder, characterized by significant volumetric changes and low stiffness (Young’s modulus ∼0.25 MPa in humans and pigs), undergoes substantial deformation during filling and emptying cycles (Tammela and Arjamaa, 1988; Van Mastrigt, 2002). Porcine bladders, closely resembling human bladders in biomechanical properties, are ideal for research purposes. Previous bladder monitoring sensors have explored various modalities, but each presents significant limitations (Supplementary Table S1). For instance, several approaches provide only an indirect inference of bladder volume. Accelerometer-based methods are invasive and infer volume from wall behavior rather than direct measurement (Weydts et al., 2018), while implanted piezoelectric (Majerus et al., 2017)and MEMS sensors (Clausen et al., 2017) are similarly invasive and measure pressure, not the direct mechanical strain of the bladder wall. Other modalities like TENG are often limited by low sensor output and high internal impedance (Arab Hassani et al., 2018). Capacitive sensors, which are closely related to our sensing mechanism, enable wireless readout options but face practical challenges. They are often passive and require precise antenna alignment with an external reader, which complicates practical use (Stauffer et al., 2018). Furthermore, studies with capacitive sensors have often been limited to phantom models, demonstrating the need for an external reader and not yet progressing to *in vivo* validation (Cao et al., 2011). Resistive sensors, while offering high compliance, also present critical limitations. Some are highly invasive and restricted to discrete, low-resolution (non-continuous) measurements (Kim et al., 2018), while others suffer from significant signal drift and performance degradation after cycling (e.g., ∼19% drift after 100 cycles) (Hannah et al., 2019). Even recent advancements using stretchable resistive materials have been confined to *ex vivo* or phantom models (Marmarchinia et al., 2025) or simple benchtop-only validation, as was the case for our group’s foundational work (Jo et al., 2021). Critically, most of these technologies, particularly single-channel resistive sensors (Mickle et al., 2019), suffer from direction-dependence, making them unreliable for capturing the complex, anisotropic expansion of a curved 3D organ like the bladder. Building on these observations, we target a soft, resistive sensor on Ecoflex to match the bladder’s low modulus and large stretch, and we mitigate direction-dependence using a three-channel layout with per-channel GF normalization.

Resistive strain sensors using stretchable and low-modulus substrates are particulary suitable for bladder monitoring due to their compliance with bladder tissue and capability to measure substantial deformation. Among common stretchable substrates (e.g., PDMS, TPU, Ecoflex) (Supplementary Table S2), Ecoflex stands out with excellent stretchability (>900%) and low modulus (∼0.008 MPa). Conductive materials, such as carbon nanotube (CNT), exhibits stable electrical conductance of up to 500% strain, used with Ecoflex (Amjadi et al., 2015). Also, there are various options for the fabrication of CNT thin film, including spray coating (Amjadi et al., 2015; Park et al., 2019), spin coating (Jo et al., 2010), chemical vapor deposition (CVD) growth (Meitl et al., 2004), stamping (Meitl et al., 2004; Zhou et al., 2006), inkjet printing (Kim et al., 2014). Among them, the spray coating method is simple and low-cost. Another thing to consider is the structural features of the bladder. Since the bladder is a 3D object, the resistance change of a sensor depends on the attached direction (Pokrywczynska et al., 2014). In addition, the smooth muscle of the bladder presents anisotropic expansion during voiding, which is also an important parameter for understanding the phyiological condition of the bladder.

While previous works have explored stick-type strain sensors, including a foundational proof-of-concept of an AuCNT sensor by our group (Jo et al., 2021), these studies were confined to benchtop environments, suffered from insufficient reproducibility, and utilized single-channel sensors capable only of gross strain detection. This approach was insufficient for capturing the complex, anisotropic deformation of a 3D organ. In this study, we addressed these limitations by expanding the sensor design to three channels to enhance compatibility with bladder deformation, while maintaining the existing high sensitivity and mechanical compliance. These multi-channel sensors were then characterized using a sufficient statistical reliability on customized testing platforms in both two-dimensional (balloon) and three-dimensional (porcine bladder) models. Additionally, the sensor data facilitated the creation of a “Virtual Bladder” model, representing a preliminary step toward a comprehensive digital twin implementation. This foundational work aims to support future advancements in adaptive neuromodulation therapies and personalized bladder dysfunction management.

## 2 Experimental section/methods

### 2.1 Material and fabrication

**FIGURE 2:**
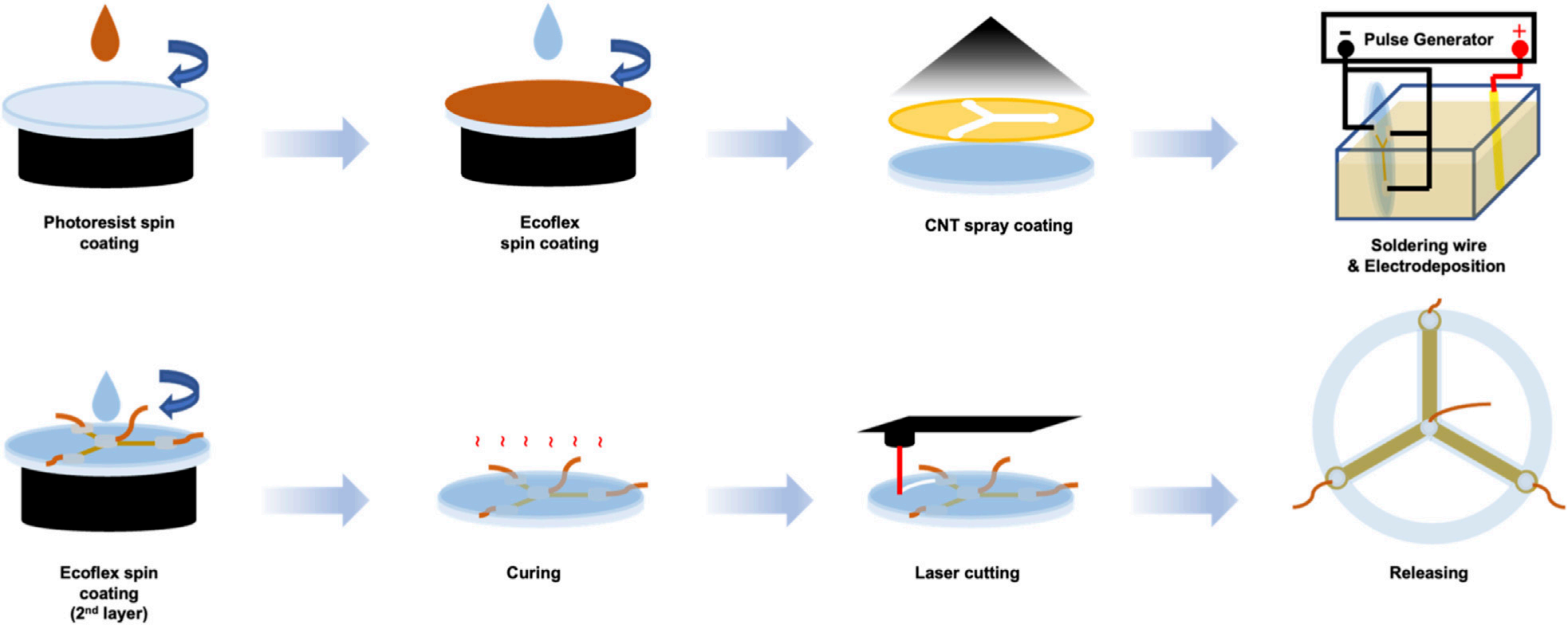
Fabrication process of the three-channel strain sensor. The sensor was fabricated by spin-coating Ecoflex on a sacrificial layer, spray-coating CNTs through a shadow mask, and electrodepositing AuCNTs to enhance sensitivity. After encapsulation and laser cutting, the final sensor was released by dissolving the sacrificial layer.

The strain sensor fabrication was executed using the following steps. First, photoresist (AZ9260, AZ Electronic Materials Ltd.) was spin-coated at 2,400 rpm for 60 s to form a 10 μm sacrificial layer on a 2 mm glass substrate. Next, Ecoflex 00-50 (Smooth-On Inc.) was spin-coated atop the photoresist. Ecoflex 00-50 parts A and B were combined in equal proportions, and air bubbles in this mixture were eliminated using a vacuum chamber. This mixture was subsequently spin-coated at 1,000 rpm for 60 s to produce a 70 μm Ecoflex substrate and cured at room temperature for 30 min. For electrode patterning, a 25 μm thick polyimide film, laser-cut into a shadow mask, was positioned over the cured Ecoflex substrate. And whole structure was placed on a hot plate at 85 °C for Carbon Nanotube (CNT) spraying. The CNT thin film was created from a CNT spray solution, comprising a commercial multi-wall carbon nanotube (MWCNT) dispersion (US Research Nanomaterials Inc., outer diameter: 20-30 nm, length: 10-30 μm, concentration: 3wt%, volume: 3 mL) diluted to 0.1wt% in 110 mL isopropyl alcohol (IPA). This solution was sonicated for 1 h at room temperature, ensuring the temperature did not surpass 35 °C. The CNT solution was sprayed onto the shadow mask at 2.4 bar pressure and 10 mL/min flow rate. Post-spraying, the polyimide mask was removed, the Ecoflex cleaned with IPA, and the structure thermally annealed in a convection oven at 150 °C for 30 min. Copper wire connections were made using silver paste and encapsulated with silicone elastomer (Kwik-sil, World Precision Instruments) (Figure 2).

### 2.2 AuCNT coating

**FIGURE 3:**
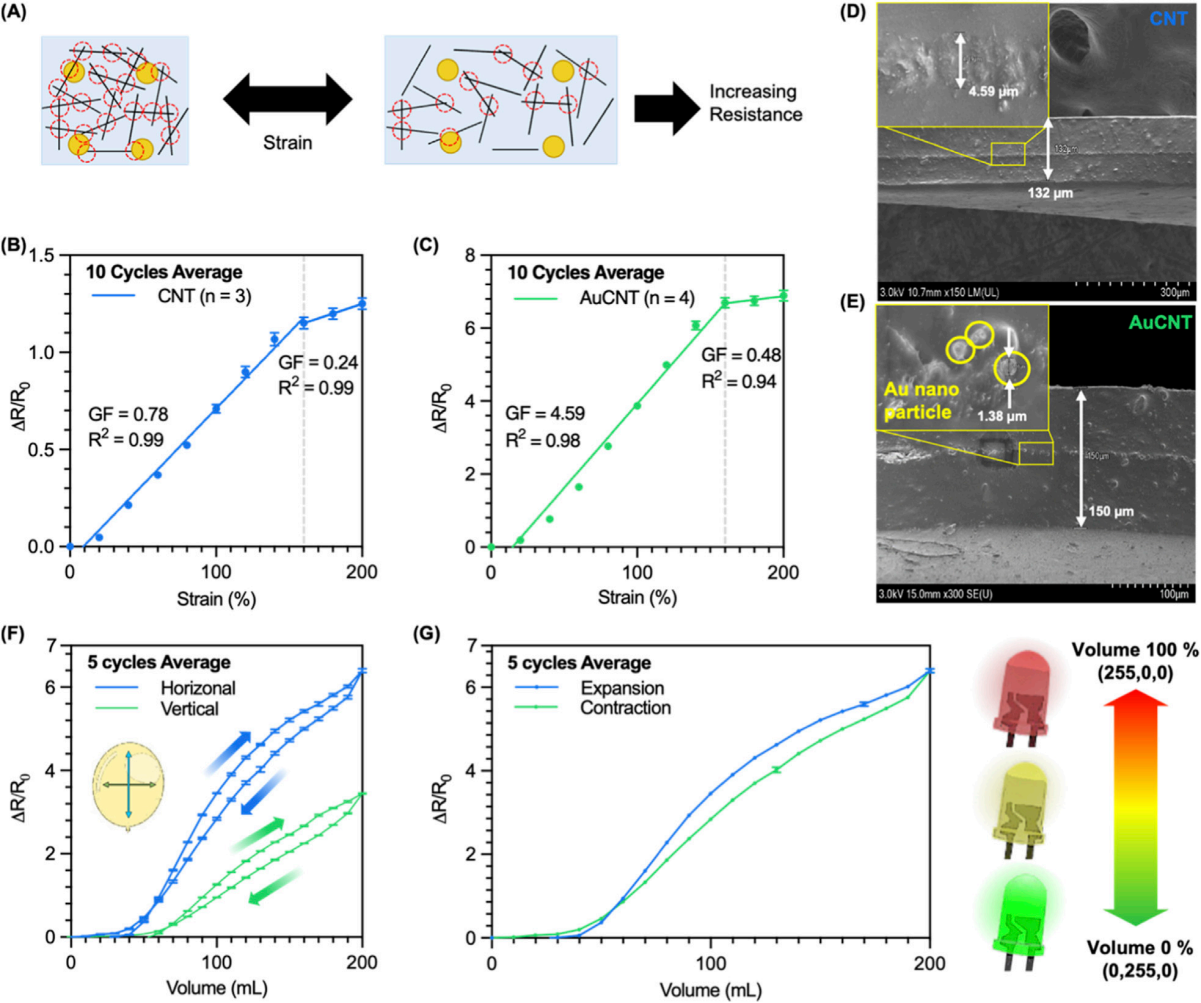
Comparison of CNT sensor and AuCNT sensor. **(A)** A schematic diagram of simple sensing mechanism of the AuCNT sensor during applying the strain. **(B)** The relative resistance changes of the CNT sensor, **(C)** and the AuCNT sensor depending on the strain change. **(D)** The SEM images (cross-section) of the CNT strain sensor, **(E)** and the AuCNT strain sensor. **(F)** Relative resistance change of the sensors according to the change in the balloon volume. **(G)** Relative resistance change applied to the monitoring system using RGB-LEDs.

To enhance sensor sensitivity, AuCNT composites were electrodeposited onto the CNT film (Figure 3A). An electrodeposition solution was prepared by dispersing short MWCNT powder (US Research Nanomaterials Inc., outer diameter: <7 nm, length: 0.5–2 μm) at 1 mg/mL concentration into commercial gold plating solution (TSG-250, Transene Company Inc.) and sonicating it for 1 h (Lee et al., 2016). Subsequently, a gold wire (anode) and the sensor (cathode) were attached to a pulse generator and placed within a 3D-printed frame, into which the electrodeposition solution was filled. Electrodeposition proceeded via monophasic pulses (1.5 V, 1 Hz, 50% duty cycle) for 8 min. A second Ecoflex layer (70 μm) was then spin-coated and cured at 70 °C for 2 h. Finally, the sensor was shaped by laser-cutting, and the AuCNT sensor was finalized by dissolving the sacrificial layer with acetone.

### 2.3 Strain-resistance measurement

A strain-resistance measurement platform was developed for the basic performance evaluation of the strain sensor. For this system, Arduino, a DC motor, and infrared sensors were used. And the law of voltage division was used to measure the resistance of the strain sensor (Supplementary Figure S1A). 100 kΩ resistor was used as a detecting resistor. Considering the input signal noise of the Arduino, 200 signals were averaged when measuring resistance at one time. A motor and an infrared sensor (GP2Y0A41SK0F), and a motor driver (L298N) were used to control the strain of the sensor (Supplementary Figure S1B). Using this platform, the resistance changes were repeatedly measured within 200% strain over 40 cycles. Resistance was measured every 20% step at 200% strain per minute. The performance of each sensor channel was specified as an average of measurements made in 10 cycles.

### 2.4 Volume-resistance measurement

Volume-resistance measurement platform was developed to measure the resistance characteristics according to the volume using Arduino, pumps, and flow sensors. Flow-in and flow-out to change the volume of the object, such as a balloon and the bladder, was implemented by using two water pumps (HS-WATER PUMP IV), two solenoid valves (HDW-2120), and two flow sensors (YF-S401). Flow-in/out lines and the balloon were connected using a T-shaped tube. And two flow lines were connected in parallel to the output terminals of the motor driver (L298N). And when the amount of water measured in the flow sensor reached the desired level, the Arduino controlled the motor driver to change flow-in and flow-out (Supplementary Figure S1C). The resistance calibration and the measurement method are the same as the strain-resistance measurement platform. In this system, the water flow rate is 10 mL/s. Using this platform, the resistance changes were repeatedly measured within 200 mL with 10 mL/s of water flow rate over 5 cycles, and the resistance was measured at every 10 mL step. The performance of the sensor was specified as an average of the 10 measurements.

### 2.5 Calculation of the relative expansion at channel and x-y coordinate

To quantify the actual stretched length across all sensors, relative expansion was calculated. Relative expansion was calculated as the resistance change divided by the GF, and was initialized to 1 by adding 1 (Equation 1).

$$Relative\;expansion\;\left(RE\right)=\frac{{\Delta R}_{n}/R_{0,n}}{{GF}_{n}}+1\;\left(n:\text{channel number}\right)$$ (1)

Furthermore, the sensor is configured as a triaxial array (Supplementary Figure S6A), and the relative expansion was transcribed onto the x-y coordinate axes to calculate the relative expansion along the x-y coordinate axes (Equations 2–5).

$${RE}_{+x}={RE}_{ch2}\cos30{}^{\circ}$$ (2)

$${RE}_{-x}={RE}_{ch3}\cos30{}^{\circ}$$ (3)

$${RE}_{+y}={RE}_{ch1}$$ (4)

$${RE}_{-y}=\frac{{RE}_{ch2}\sin30{}^{\circ}+{RE}_{ch3}\sin30{}^{\circ}}{2}$$ (5)

### 2.6 Virtual bladder modeling

Virtual bladder modeling: The measurement values of the 3-channel strain sensor were used to implement the virtual bladder for digital twin. One of the limitations of the developed strain sensor is that it is difficult to define the height of the entire bladder and the diameter of the cross section to define the volume of the virtual bladder because the shape of the sensor is not in the form of monitoring the strain of the entire bladder. To solve this problem, a virtual distance was defined for virtual bladder implementation through measurement values. The height and radius of the virtual distance were calculated through the measured value (relative expansion), and the y-axis of the actual measured value (relative expansion) was defined as the measured height in the x-y coordinates, and half of the length of the x-axis was defined as the measured radius (Equations 6, 7).

$$reR=\frac{{RE}_{+x}+{RE}_{-x}}{2}$$ (6)

$$reH={RE}_{+y}+{RE}_{-y}$$ (7)

The initial values of the height and radius of the virtual distance were defined by multiplying the measured height and radius ( reR , reH ; Relative expansion of the sensor transcribed into x-y coordinates) measured at 100 mL by a constant such that the volume of the bladder digital twin was 100 mL (Supplementary Figure S2A; Equation 8). The virtual distance defined in the virtual bladder does not represent the entire length of the virtual bladder, and since the real bladder expands anisotropically rather than linearly, the virtual distance for subsequent volumes was defined by the sum of the multiplying the previous value by a constant and multiplying the measured value by a constant (Equation 9). The virtual distance refers to the height ( $\left.vH\right)$ and radius vR of the virtual bladder and considering that the sensor does not attach to the entire bladder, the height and radius of the virtual bladder were defined as a portion of the virtual bladder (Supplementary Figure S2B).

$$\left({vH}_{0},{vR}_{0}\right)=5.3\times \left({reH}_{0},{reR}_{0}\right)$$ (8)

$$\left({vH}_{n},{vR}_{n}\right)=0.9\times \left({vH}_{n-1},{vR}_{n-1}\right)+1.2\left({reH}_{n},{reR}_{n}\right)$$ (9)

The shape of the virtual bladder was defined as a solid of revolution of a curve, and the curve was defined through a virtual distance. The curve was defined with a total of three intervals, the first interval was defined as a quadratic function (Equations 10, 11), the second interval was defined as a square root function (Equations 12, 13), and the last interval was defined as the translation of the quadratic function (Equation 14).

$$A=\frac{h^{2}}{4\times r}$$ (10)

$$f_{1}\left(x\right)=\frac{x^{2}}{A}$$ (11)

B=3+0.06r (12)

$$f_{2}\left(x\right)=B\sqrt{x-0.5vH}+vR$$ (13)

$$f_{3}\left(x\right)=-\frac{{\left(x-1.5vH\right)}^{2}}{vH}+B*\sqrt{vH}+vR$$ (14)

The volume of the bladder digital twin was defined through the volume of the solid of revolution of the defined curve, and the interval and defined volume for each curve are as shown in Equations 15, 16. Rhinocore 6 and grasshopper (Robert McNeel & Associates) were used to create the bladder 3D model.

$$F=1.5vH+\sqrt{vH*\left(B*\sqrt{vH}+vR\right)}$$ (15)

$$V=\pi \;\int_{0}^{\frac{h}{2}}{f_{1}\left(x\right)}^{2}\;dx+\pi \;\int_{\frac{h}{2}}^{\frac{3h}{2}}{f_{2}\left(x\right)}^{2}\;dx+\pi \;\int_{\frac{3h}{2}}^{F}{f_{3}\left(x\right)}^{2}\;dx$$ (16)

## 3 Result

### 3.1 Characterization of stick-shaped AuCNT strain sensor in strain-resistance measurement platform

Flexible and stretchable strain sensors were optimized depending on the fabrication conditions, such as Ecoflex spin coating, CNT spray coating, and AuCNT deposition, and a detailed description is in the experimental section. Firstly, a stick-shaped strain sensor with a length of 20 mm and a width of 2 mm was fabricated to optimize the AuCNT deposition. Two types of sensors were fabricated for comparison; only CNT deposited and AuCNT deposited sensors, and every fabrication processes are the same except for the additional deposition of AuCNT. Then, the resistance characteristics of the two sensors were measured under strain from 0% to 200%. To measure repeatedly and reliably, the strain-resistance measurement platform (Supplementary Figure S1B) and the volume-resistance measurement platform (Supplementary Figure S1C) were developed. The detailed explanations are in the experimental section.

The strain sensor with only CNT showed a resistance value of 117 ± 46 kΩ (n = 3) at 0% strain and 261 ± 92 kΩ (n = 3) at 200% strain. While the AuCNT composited strain sensor showed a resistance value of 58 ± 14 kΩ (n = 4) at 0% strain and 450 ± 77 kΩ (n = 4) at 200% strain. The sensitivity of a strain sensor is typically calculated by the relative resistance change (R- R0)/R0, where R0 and R represent the resistance before and after a strain, respectively. The relative resistance change (ΔR/R0, where ΔR = R-R0) of the CNT strain sensor was 1.25 ± 0.16 (n = 3) (Figure 3B), while that of the AuCNT strain sensor was 6.89 ± 0.93 (n = 4) at 200% strain (Figure 3C). The gauge factor (GF) of the device is defined as GF=(ΔR/R0)/ε, where ε is the strain. The strain sensors showed different GFs at different strains. The GF of the CNT strain sensor showed 0.78 at a relatively lower strain of 0%–160% and 0.24 at a relatively higher strain of 160%–200% (Figure 3B). While the GF of the AuCNT strain sensor showed 4.59 at the lower strain and 0.48 at the higher strain (Figure 3C). The GF of the AuCNT strain sensor increased approximately 5.9 times than that of the CNT strain sensor at the lower strain of 0%–160%. However, at 200% strain, the SNR of the CNT strain sensor and AuCNT strain sensor were 17.85 dB and 17.47 dB, respectively, confirming that there was no significant change in SNR before and after coating (Equation 17). These results suggest that the AuCNT strain sensor can be used for monitoring strain in the wide range (0%-160%) with high sensitivity.

$$SNR=20\log_{10}\frac{mean\;of\;relative\;resistance\;change}{S.D.\;of\;relative\;resistance\;change}$$ (17)

The AuCNT composite sensor demonstrated a significantly enhanced gauge factor, approximately 5 times higher than the CNT-only sensor. This performance improvement is supported by direct physical evidence from cross-sectional SEM imaging (Figures 3D,E) and is attributed to the alteration of the sensor’s conductive network. The baseline CNT-only sensor mechanism is dominated by the tunneling effect between adjacent CNTs. As the substrate stretches, the inter-CNT distance increases, which exponentially increases the tunneling resistance. The SEM images confirm the structural changes resulting from the gold deposition. While the overall sensor thicknesses were comparable (130 µm for CNT vs. 126 μm for AuCNT), the conductive film itself was significantly thicker in the AuCNT sensor (8.31 µm) compared to the CNT-only film (4.89 µm). As shown in Figure 3E, this additional material consists of nano-sized Au islands, with a maximum size of about 1 μm, formed on the CNT film surface. These Au islands function as conductive bridges, creating new, parallel pathways (e.g., CNT-Au-CNT and Au-Au). This new network enhances the electrical contact between CNTs, which directly explains the significantly lower initial resistance of the AuCNT sensor (58 kΩ) compared to the CNT sensor (117 kΩ). Crucially, these new Au-mediated junctions are also highly sensitive to strain. The AuCNT network thus possesses a higher density of strain-sensitive tunneling junctions (Wang et al., 2022). When the sensor is stretched, the widespread and efficient disconnection of these numerous pathways—in addition to the standard CNT-CNT separation—results in a much larger and sharper collective resistance change, leading to the significantly enhanced gauge factor (Madhavan, 2024).

### 3.2 Characterization of stick-shaped AuCNT strain sensor in balloon-model with volume-resistance measurement platform

The characterized AuCNT strain sensor was also applied in the balloon-model along with the volume-resistance measurement platform developed to investigate volume monitoring before applying it to porcine bladders. Since the output of the sensor is different depending on the direction in which the sensor is attached, the strain sensor is attached to the balloon wall in two directions, horizontally and vertically, to measure the resistance characteristics according to the volume, respectively. At 200 mL of the volume, the total lengths of the strain sensor stretched were 38 mm (90% strain) horizontally and 32 mm (60% strain) vertically (Supplementary Figure S3). The resistance change rates at 200 mL in the two cases were 7.40 ± 0.11 (5 cycles average) horizontally and 4.45 ± 0.04 (5 cycles average) vertically (Figure 3F). As a result of the repeated measurements, the standard deviation of the values measured in 5 cycles was within 2% of the average value, showcasing that the fabricated sensors can measure the volume of the balloon repeatedly and reliably.

To track the volume of the balloon using the developed sensor, RGB LEDs were connected to the sensor, and the color change of the LED according to the volume of the balloon was investigated (Figure 3G). As shown in Supplementary Video S1, when the volume of the balloon increases, the color of the LEDs changes from green to red, estimating the volume of the balloon. This result indicates that the developed sensors with the measurement system enable measuring and tracking the volume change in the 3D balloon-model.

### 3.3 Characterization of stick-shaped AuCNT strain sensor in porcine bladder (*ex-vivo*)

The developed sensor was applied to the porcine bladder. The sensor was attached to the wall of the extracted porcine bladder to measure the resistance characteristics according to the volume. The sensor was attached horizontally to the bladder wall with surgical sutures and silicone elastomer to demonstrate the maximum resistance change in the bladder, and the stretching of the sensor was stable the volume increase of the bladder at a volume of 600 mL, known as the maximum volume of the porcine bladder. When the bladder volume was 600 mL, the length of the stretched sensor was 50 mm long, estimating 150% strain applied to the sensor in the bladder wall (Supplementary Figure S4A). While the bladder volume expands to 600 mL, the relative resistance change rate increased to 11.75, which is a higher value than the result in the 2D environment at the 150% strain (Supplementary Figure S4B). Also, the relative resistance change in the balloon-model is slightly higher than in the 2D model, which may be due to the fact that the pressure from the curvature of the balloon or the bladder affects the relative resistance changes, indicating that 3D models, including bladders, should consider this curvature-based pressure aspect during the measurement.

### 3.4 Characterization of three-channel AuCNT strain sensor in balloon-model

Based on the previous results, the curvature from the 3D target model affected the results measured in the 2-D model. Thus, measuring the relative resistance change according to the volume in 3D models is important. Furthermore, the strick-shaped sensor has limitations in that the output of the sensor will differ depending on the attached direction on a bladder wall. Accordingly, a three-channel strain sensor was suggested to reduce the dependence on the attached direction and to track the expansion of the bladder (Supplementary Figure S5A). Each channel has the electrical connection independently and is coated in AuCNT at once during the fabrication process. The width of each channel is the same as the stick-shaped sensor of 2 mm, but the length is 10 mm, shorter than that of the stick-shaped sensor, reducing sensitivity to the pressure caused by the curvature (Supplementary Figure S5B).

**FIGURE 4:**
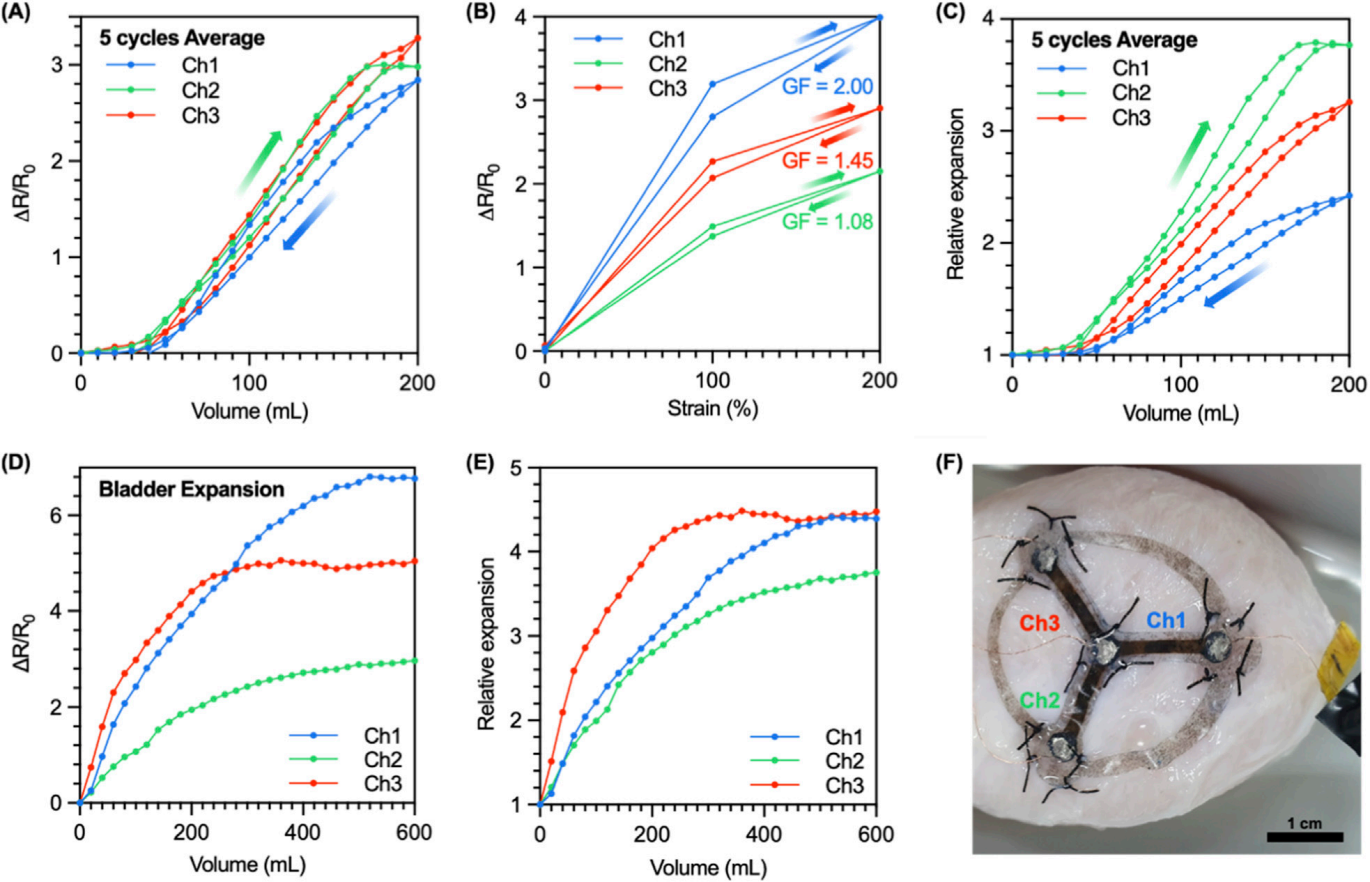
Volume-Resistance characteristics and expansion tracking of the sensor on a balloon-model and porcine’s bladder **(A)** The result of the relative resistance change according to the balloon volume changes. **(B)** The result of the relative resistance change depending on the strain and the gauge factor at 200% strain of each channel. **(C)** Relative expansion of the sensor, calculated by each gauge factor. **(D)** The resistance change rate according to the volume. **(E)** Ex-vivo test of the sensor for detecting of relative expansion. **(F)** The sensor sutured on the bladder wall.

The sensor was attached to the center of the balloon, with channel-1 facing upwards for the volume-resistance measurement. Also, the measurement was conducted in the range of 200 mL as shown in Supplementary Video S2. The relative resistance change at the maximum volume of 200 mL was 2.84 ± 0.10 (5 cycles average), 2.98 ± 0.01 (5 cycles average), and 3.28 ± 0.11 (5 cycles average), respectively (Figure 4A). However, each channel has different GFs due to the limitation of the electrodeposition method for the three-channel coating at once. The GF of each channel was measured using the strain-resistance measurement system to normalize each channel. The GFs of each channel are 2.00, 1.08, and 1.45, respectively (Figure 4B). Then, relative expansion (RE) was calculated based on Equation 1 for volume tracking, where RE is 1 at 0% strain.

Relative expansion was calculated according to the volume changes. The relative expansion values at maximum volume (200 mL) for each channel were 2.42, 3.77, and 3.26, respectively (Figure 4C). The lengths of each channel at 200 mL were 16 mm (60% strain), 25 mm (150% strain), and 22 mm (120% strain) (Supplementary Figure S5C). Both results match well with the ratio among the three channels showing a trend in channel 2 > channel 3 > channel 1. This result indicates that the three-channel sensor enables the measurement of the volume of a 3D model and even expansion direction by calculating relative expansion based on the resistance characteristics.

### 3.5 Characterization of three-channel AuCNT strain sensor in porcine bladder (*ex-vivo*)

The characterized sensor was applied to the porcine bladder to track the volume and expansion. The three-channel strain sensor was attached using the surgical suture and the silicone elastomer on the wall of a porcine bladder (Figure 4F). The measurements were conducted in the range of 600 mL every 20 mL. The relative resistance change for each channel were 6.77, 2.96, and 5.05 at the maximum volume of 600 mL (Figure 4D). The relative expansion was calculated from the measured result and the previously calculated gauge factor. The relative expansion values at the maximum volume for each channel were 4.39, 3.75, and 4.48, respectively (Figure 4E). And the lengths of each channel were 32 mm (220% strain), 25 mm (150% strain), and 36 mm (260% strain), respectively (Supplementary Figure S6). This result also showed the same tendency as channel 3 > channel 1 > channel 2, as the result in the balloon-model.

### 3.6 Expansion tracking of the three-channel AuCNT strain sensor

**FIGURE 5:**
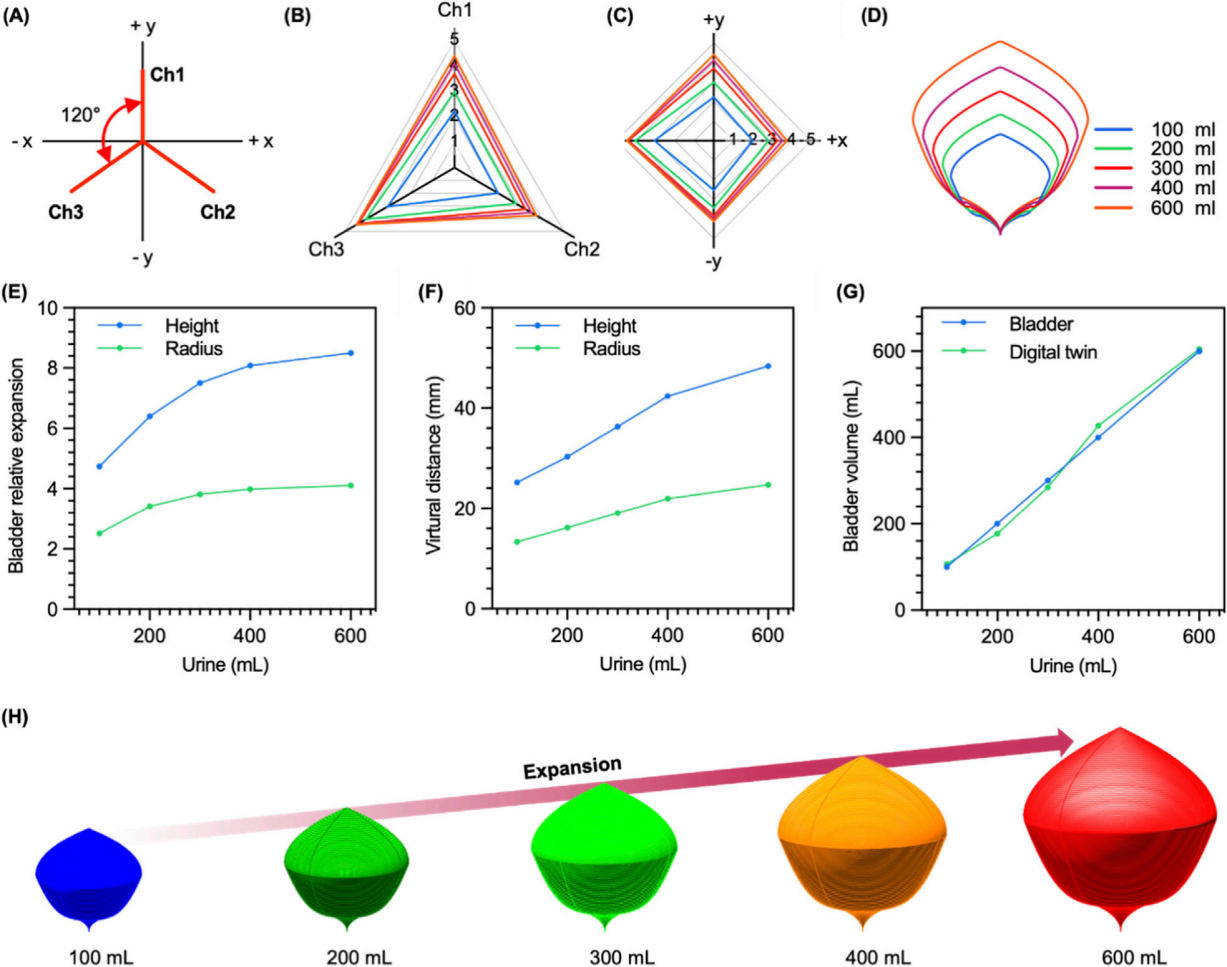
Virtual bladder of porcine’s bladder. **(A)** The sensor structure projected in x-y plane. **(B)** Expansion tracking on three-channels. **(C)** Conversion to x-y coordinates. **(D)** Longitudinal section of virtual bladder for each volume. **(E)** Relative expansion of measurements value (height, radius). **(F)** The height and radius value of the virtual distance. **(G)** The volume of the virtual bladder and the volume of the real porcine’s bladder. **(H)** Volume change of virtual bladder.

Through the calculated relative expansion, the direction in which the bladder wall expands according to the bladder volume was indirectly measured. The sensor consists of three channels with an angle of 120° between each channel (Figure 5A). The related expansion is expressed in the coordinates of a two-dimensional plane with each channel as an axis (Figure 5B), which can be later converted into x-y coordinates using Equations 2–5 to express the expansion of the bladder according to the volume in a two-dimensional plane (Figure 5C).

As a result, it was shown that the volume of the porcine bladder was rapidly expanding in the –x direction up to 300 mL and, after that, uniformly in the +x and +y directions. The result indicates that the three-channel strain sensor successfully demonstrates tracking the direction of expansion according to the volume of the bladder.

### 3.7 Strain error of the three-channel strain sensor

A comparison was conducted to confirm that the three-channel strain sensor was more accurate in volume tracking than the stick-type (1-channel) strain sensor. The measurement errors in strain for the stick-type and three-channel strain sensor were calculated using Equation 18. As a result, the error of the stick-shaped strain sensor was 39.19%, and the errors of the three-channel strain sensor were calculated as 27.02%, 33.17%, and 19.69%, respectively, for each channel. These results demonstrate that the three-channel strain sensor is more accurate in tracking bladder volume than the stick-shaped strain sensor.

$$\left(1-\frac{L/L_{0}}{\frac{\Delta R/R_{0}}{GF}+1}\right)\times 100\;\%$$ (18)

### 3.8 Virtual bladder modeling via three-channel AuCNT strain sensor

To obtain the virtual distance for realizing the bladder digital twin, the length of the y-axis of the RE moved to x-y coordinates was defined as the height of the RE, and half of the length of the x-axis was defined as the radius of the relative expansion (Figure 5E). The height and radius of the virtual distance were calculated with the components defined in relative expansion (Figure 5F). The shape of the longitudinal section and the shape of the digital twin of the bladder was defined according to the volume of the virtual bladder by generating the envelop curve through a function defined for the radius of the cross section of the virtual bladder (Figures 5D,H). Compared to the actual volume, The errors according to each volume were 7.15%, 11.55%, 5.19%, 6.74%, and 0.71% from 100 mL to 600 mL (except 500 mL), with an average of 6.27% (Figure 5G).

## 4 Discussion

The significance of this work lies in its demonstration of two foundational pillars required for a viable digital-twin-based bladder control system. First, the developed AuCNT-Ecoflex strain sensor demonstrates considerable potential, primarily owing to its novel, flexible architecture. The sensor exhibited a gauge factor (GF) 5.9 times higher than conventional CNT-based counterparts. This high sensitivity is paramount for accurately tracking the subtle volumetric fluctuations characteristic of bladder function and provides the high signal-to-noise ratio necessary to detect the onset of bladder filling, capturing subtle wall strains long before they become large. Second, by implementing a three-channel design, this work overcomes a critical limitation of single-channel systems: the inability to account for the bladder’s anisotropic expansion. This multi-channel sensing approach yielded a more faithful representation of volume changes up to 600 mL in the porcine model and established the feasibility of translating complex, anisotropic strain data into a coherent 3D volume estimation. The data stream from this system enabled the construction of a preliminary “Virtual Bladder” (Figure 5), which serves as an important proof-of-concept for a more sophisticated digital twin. The validity of our measurement platforms was confirmed using both two-dimensional and three-dimensional models, affirming the sensor’s capacity to translate mechanical strain into reliable volumetric data.

To contextualize the potential of our strain-based approach, it is valuable to compare it with current bladder monitoring standards. The most common non-invasive method, portable 3D ultrasound (e.g., BladderScan), offers an immediate, ‘snapshot’ volume estimation at the point-of-care. However, it is not suitable for continuous, real-time monitoring and its accuracy can be limited. Studies have shown significant error margins, with some devices exhibiting average overestimations of +17.5% and others underestimations of −6.3% compared to the actual catheterized volume (Brouwer et al., 2018). Another emerging wearable technology, bio-electrical impedance analysis (BIA), shows promise for continuous, non-invasive tracking and has demonstrated high correlation coefficients (e.g., 96.7%) with bladder volume in preliminary studies (Duan and Jin, 2025). However, BIA measurements are inherently non-specific, as the electrical impedance path is influenced by all tissues and fluids between the electrodes, making the signal susceptible to motion artifacts and changes in overall body fluid composition. Our AuCNT strain sensor, while currently demonstrated ex-vivo, represents a different paradigm. As a potential future implantable device, it would offer continuous, real-time data based on direct mechanical deformation of the bladder wall, rather than inferred acoustic (US) or electrical (BIA) properties. This direct measurement could provide unparalleled fidelity, free from the non-specificity of BIA or the intermittent nature of ultrasound. Compared to other implantable technologies like MEMS pressure sensors (Buchwalder et al., 2023), our device measures strain (deformation), not pressure, which may provide more direct insights into wall stress and the anisotropic expansion patterns critical for a digital twin model.

For this technology to progress toward clinical application, however, several technical and practical limitations require careful consideration. The virtual bladder model, while a valid proof-of-concept, is still in its nascent stages, lacking the multimodal data integration and anatomical specificity needed for true patient-specific modeling. It also cannot yet predict factors like tissue elasticity or stress distribution. A key technical limitation lies in the calibration of its defining parameters. The scaling factor (5.3) used to match the initial virtual volume to 100 mL, as well as the update weights (0.9 for the previous value and 1.2 for the measured value) in Equation 9, were empirically optimized based on a single ex-vivo porcine bladder dataset. Consequently, these constants are not universally applicable and would almost certainly require re-calibration for different subjects (inter-subject variability) or even varied sensor placement on the same subject. This highlights that our current model is a proof-of-concept for volume tracking rather than a generalized model for volume estimation. We also observed a notable variance in the baseline GFs across the three channels (2.00, 1.08, and 1.45, respectively, as shown in Figure 4B). This variability is a direct consequence of limitations in our current fabrication process, specifically the AuCNT electrodeposition. However, this variance does not compromise the sensor’s utility; on the contrary, it is precisely why a per-channel GF normalization, as described in Equation 1, is a necessity for this type of multi-channel sensor. By calculating the relative expansion (RE) for each channel independently, our model successfully compensates for this fabrication-induced heterogeneity, demonstrating the robustness of our approach.

Substantive obstacles also remain at the practical and translational level. Regarding sensor positioning, while the 3-channel design with GF normalization addresses local directionality, the global position and orientation of the sensor array on the bladder surface remain critical. The results presented demonstrate reliable volume estimations assuming a fixed sensor position. A significant limitation remains: if the sensor is detached and re-attached, or if its initial position shifts substantially, the established relationship between the measured strain vectors and the virtual bladder geometry would be invalidated. This dependency on a fixed initial calibration poses a challenge for long-term implantable solutions. Furthermore, the current sensor attachment method, utilizing surgical sutures, is inherently invasive and suitable only for the ex-vivo model or acute in-vivo studies. For any chronic clinical application, a robust, minimally invasive, and biocompatible fixation method is essential. Moreover, this study did not evaluate the sensor’s long-term performance and stability while fully submerged in a physiological liquid environment. Characterizing the sensor’s electrical drift, the long-term integrity of the Ecoflex encapsulation, and the potential for biofouling is a critical next step. The need to establish a scalable, cost-effective fabrication process also remains.

## 5 Conclusion

This research has introduced a highly sensitive and stretchable AuCNT-Ecoflex strain sensor system capable of monitoring bladder volume. We have shown that this system can effectively map the anisotropic expansion of the bladder and provide real-time visual feedback on volumetric status. While the path to clinical implementation requires addressing key challenges in manufacturing, long-term stability, and biocompatibility, future work should be directed toward resolving these identified limitations. To address the calibration dependency, future studies must involve larger, more diverse datasets to develop a more generalized, subject-independent modeling approach or, perhaps more practically, a robust and streamlined calibration protocol for clinical translation. We must also explore alternative implantation strategies, such as the use of biocompatible adhesives, refined manufacturing protocols, and comprehensive biocompatibility studies to ensure long-term measurement fidelity. The ultimate translation of this ex-vivo system would involve this sensor acting as the primary feedback element in a closed-loop neuromodulation system. By providing continuous, high-fidelity data on both the magnitude (from high GF) and direction (from 3D tracking) of bladder deformation, this sensor could enable an adaptive neuromodulation algorithm to intervene only when needed, moving beyond simple detection and toward a truly autonomous, patient-specific therapeutic device for neurogenic bladder dysfunction. The presented framework represents a foundational step toward developing more sophisticated, personalized neuromodulation therapies for individuals with neurogenic bladder dysfunction.

## Data Availability

The original contributions presented in the study are included in the article/Supplementary Material, further inquiries can be directed to the corresponding author.
