# Supplementary material for: Toward virtual bladder: real-time bladder volume monitoring with flexible AuCNT strain sensors
Source: Front Bioeng Biotechnol. 2026 Jan 5;13:1717576. doi: 10.3389/fbioe.2025.1717576 (PMC12813022; doi:10.3389/fbioe.2025.1717576)
Supplement: Supplementary file 1 [file Supplementaryfile1.docx]

Supplementary Material

# Supplementary Figures and Tables

## Supplementary Figures

**
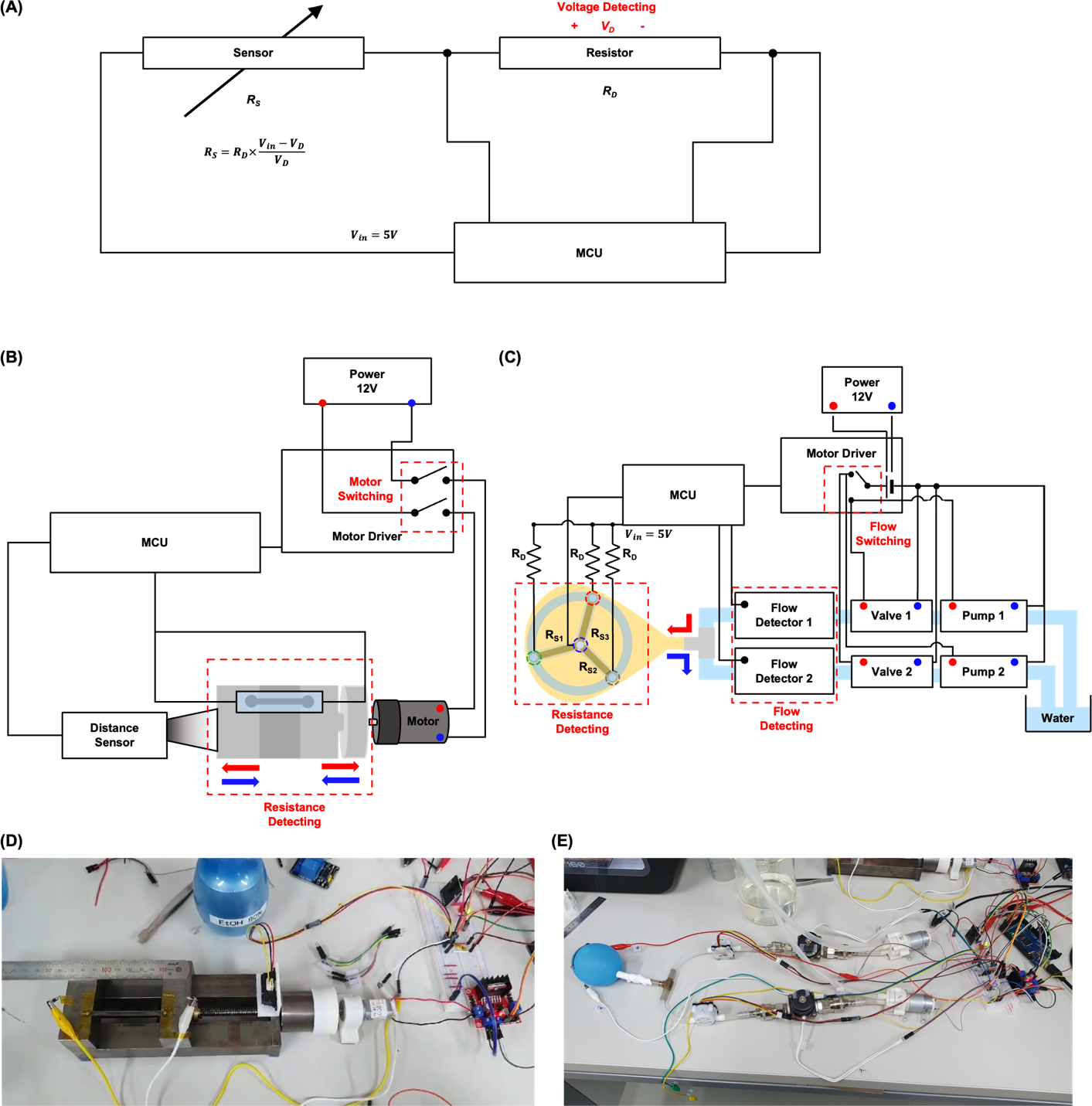
**

**Supplementary Figure 1.** Schematic diagram of measurement system and platform. (A) Resistance measurement system via voltage division law. (B, D) The strain-resistance measurement platform. (C, E) The volume-resistance measurement platform.

**
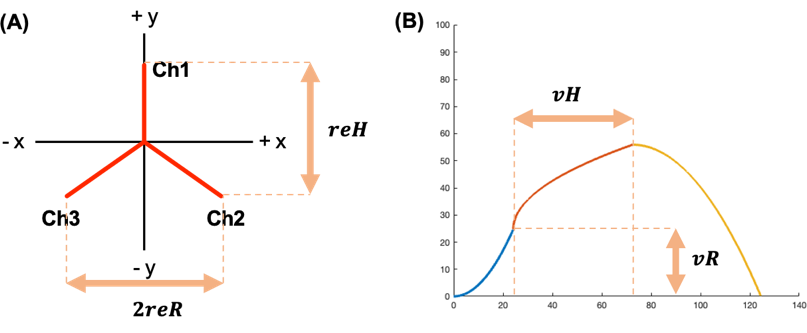
**

**Supplementary Figure 2.** (A) Diagram of the height and diameter of the distance measured in the x-y coordinate example. (B) Diagram of the area corresponding to the virtual distance (height, diameter) in the implemented virtual bladder. The blue graph represents the first area (f_1_ in the manuscript), the red graph represents the second area (f_2_ in the manuscript), and the yellow graph represents f_3_ in the manuscript.


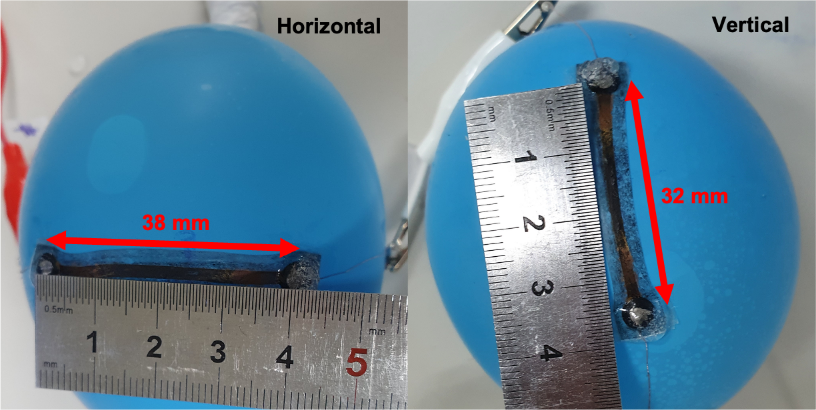


**Supplementary Figure 3.** Pictures of the stick-shaped sensor attached to the balloon horizontally and vertically at 200 mL, respectively.


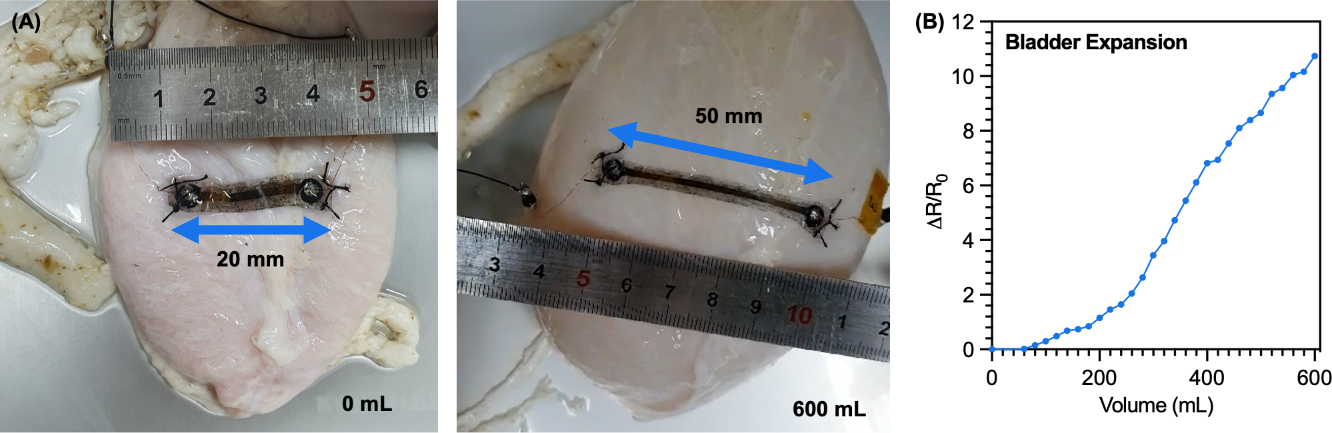


**Supplementary Figure 4.** (A) Pictures of the strain sensor sutured on the wall of the porcine bladder with the length change at the volume of 0 mL and 600 mL, respectively. (B) The result of the relative resistance change of the sensor according to the bladder volume changes.


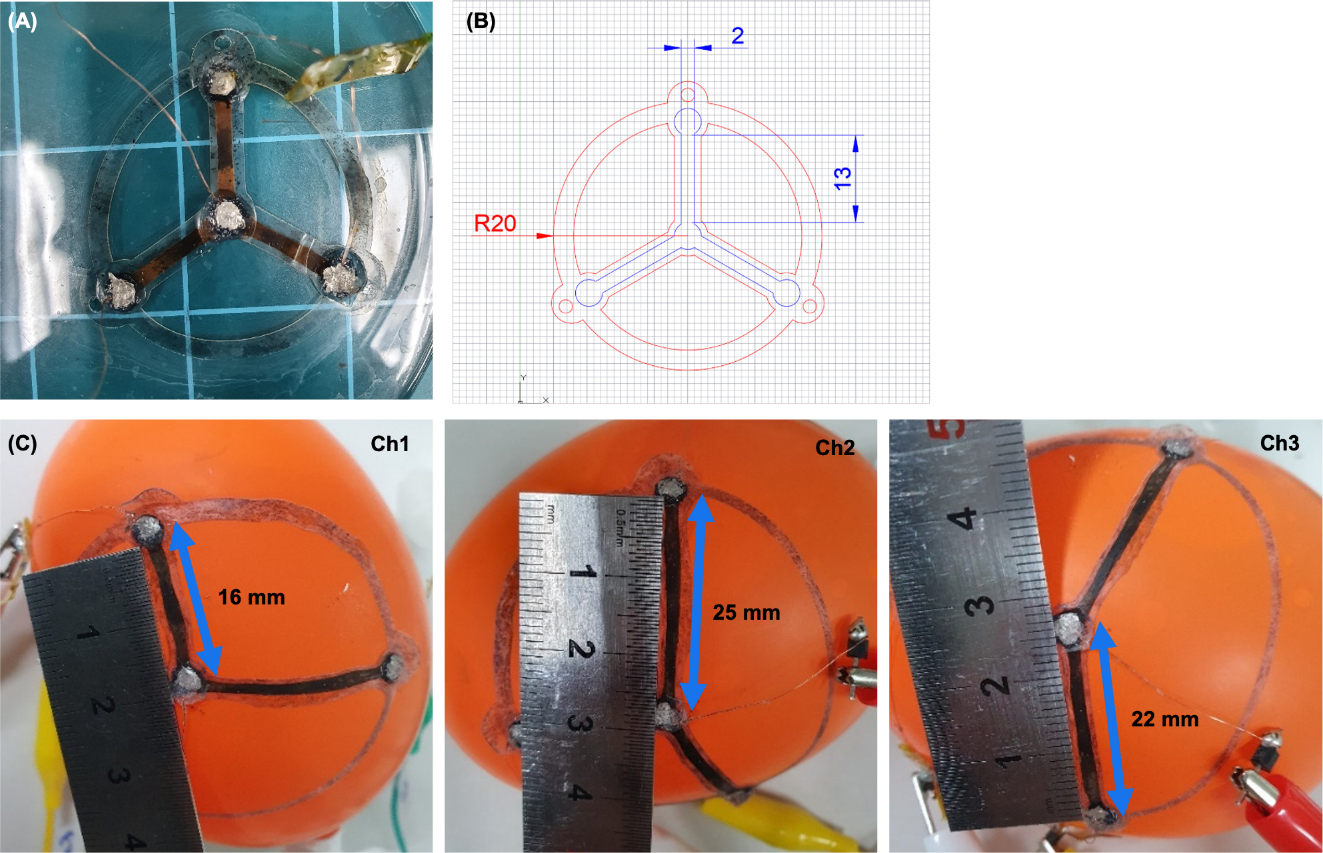


**Supplementary Figure 5.** (A) Three-channel bladder strain sensor. (B) Sensor design drawing. (C) Volume-Resistance characteristics and expansion tracking of the sensor on a balloon-model and the length of each channel at the volume of 200 mL.

**
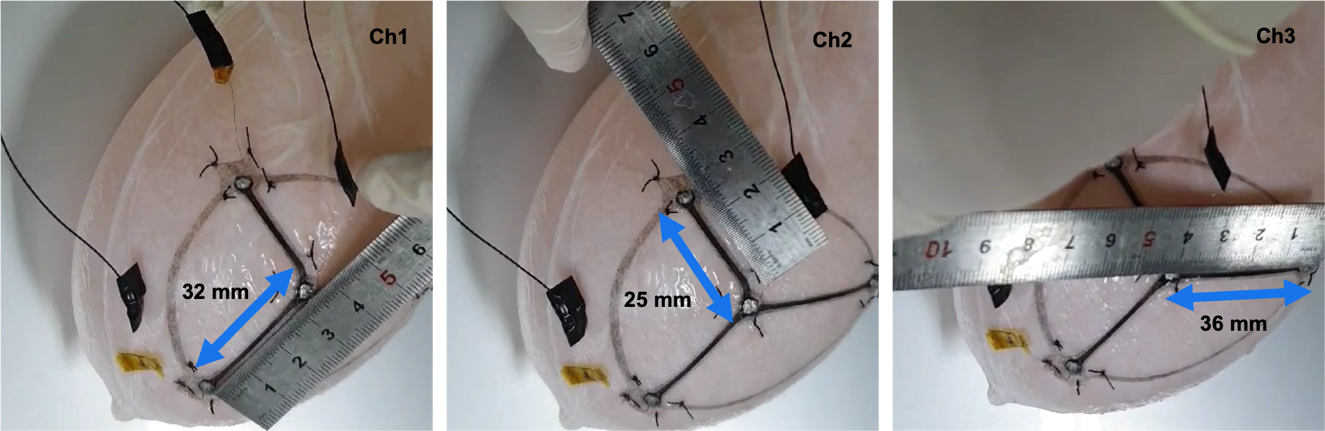
**

**Supplementary Figure 6.** The length of each channel at volume of 600 mL. Bladder expansion tracking according to the volume.

## Supplementary Table

1. State of the art of the bladder monitoring sensor

| **Paper** | **Type** | **Target** | **Sensing Range** | **Sensitivity** | **Limitation** |
| --- | --- | --- | --- | --- | --- |
| Kim et al., 2018 | Resistive (Contact Switch) | Porcine (ex-vivo) | Discrete (658, 520, 436, 352 mL) | - | Low resolution (non-continuous), Calibration, Encapsulation |
| Hannah et al., 2019 | Resistive (Au on PU) | Pig  (ex-vivo) | ~ 50% strain  60 ~ 160 mL | 3.18 Ω/% (L=6mm) | Signal drift (hysteresis) (~19% after 100 cycles)  Placement Dependency |
| Mickle et al., 2019 | Resistive (Strain Gauge) | Rat (in-vivo) | - | - | Pre-Algorithm Training, Individual Calibration |
| Yan et al., 2019 | Piezoresistive & Capacitive (CNT-based) | Feline (in-vivo) | Up to 100% strain | - (High correlation reported) | Need pre-stretching, Drift, Capacitive Sensor complexity, Substrate tearing |
| Stauffer et al., 2018 | Capacitive (RLC circuit) | Bladder monitoring (concept) | 0 ~ 200 mL (ex-vivo) | - (Measures resonant frequency shift) | Sensing Threshold, Hysteresis, Wireless range |
| Weydts et al., 2018 | Accelerometer & Pressure | Rat, Larger animal models (in-vivo) | - | - | Model size Limit, Re-calibration Required, Offset drift |
| Majerus et al., 2017 | Piezoelectric (Pressure) | Feline & Canine (in-vivo) | -  (Pressure sensor) | 0.8 cm H2O | Erosion, Wireless required, Signal Damping & Drift, Small size sample |
| Clausen et al., 2017 | Piezoresistive  MEMS (Pressure) | Human | -  (Pressure sensor) | - | No absolute pressure, Sensor mismatch, Imperfect zeroing |
| Arab Hassani et al., 2018 | TENG | Rat (in-vivo) | 0 ~ 1 mL | -  (Increase voltage at a certain volume) | Encapsulation issue  Biocompatibility  Actuator limitation |
| Cao et al., 2011 | Capacitive (interdigitated) | Balloon phantom model | 390–450 mL (~1.7–4.9% strain) | ~8 Hz /% strain | Fluctuations (at real-time); phantom-only (no in vivo) |
| Marmarchinia et al., 2025 | Resistive (stretchable wrinkled metal) | Bladder phantom (in vitro) & pig bladder (ex vivo) | ~ 420 mL (Phantom)  ~ 300 mL  (Ex-vivo) | ~4.60 mL⁻¹ | Preconditioning, Hysteresis / Response time, Placement sensitivity |
| Jo et al., 2021 | Resistive | Benchtop | 200 % | GF = 3.26 | Benchtop only |
| **This work** | **Resistive (AuCNT-Ecoflex)** | **Porcine (ex-vivo)** | **~ 600 mL (~260% strain)** | **GF (Stick) = 3.768 ( ~ 200 % strain)**  **GF (3 ch) = 1.08 - 2.00** | Ex-vivo only, GF variability (requires calibration),  Low sample size, Calibration |

1. Young`s modulus and stretchability of bladder and biocompatible substrate materials

| Materials | Young`s modulus (kPa) | Stretchability (%) |
| --- | --- | --- |
| Rat bladder | 760 (Pokrywczynska et al., 2014) | - |
| Porcine bladder | 260 (Pokrywczynska et al., 2014) | 200 (Van Mastrigt, 2002) |
| Human Bladder | 250 (Pokrywczynska et al., 2014) | - |
| PDMS | 1840 (Ni and Zhang, 2017) | 130 (Wu et al., 2018) |
| Ecoflex 0050 | 83 (Ni and Zhang, 2017) | 980* |
| Ecoflex 0030 | 69 (Ni and Zhang, 2017) | 900* |
| TPU | 3340(Kanbur and Tayfun, 2018) | 331 (Kanbur and Tayfun, 2018) |

*by Smooth-On, Inc

# References

Kanbur, Y., and Tayfun, U. (2018). Investigating mechanical, thermal, and flammability properties of thermoplastic polyurethane/carbon nanotube composites. *Journal of Thermoplastic Composite Materials* 31, 1661–1675. doi: 10.1177/0892705717743292

Ni, N., and Zhang, L. (2017). “Dielectric Elastomer Sensors,” in *Elastomers*, (InTech). doi: 10.5772/intechopen.68995

Pokrywczynska, M., Jundzill, A., Adamowicz, J., Kowalczyk, T., Warda, K., Rasmus, M., et al. (2014). Is the poly (L- lactide- co- caprolactone) nanofibrous membrane suitable for urinary bladder regeneration? *PLoS One* 9. doi: 10.1371/journal.pone.0105295

Van Mastrigt, R. (2002). Mechanical properties of (urinary bladder) smooth muscle. *J Muscle Res Cell Motil* 23, 53–57.

Wu, S., Peng, S., and Wang, C. H. (2018). Stretchable strain sensors based on PDMS composites with cellulose sponges containing one- and two-dimensional nanocarbons. *Sens Actuators A Phys* 279, 90–100. doi: 10.1016/j.sna.2018.06.002
